# Emerging Infectious Diseases and One Health: Implication for Public Health

**DOI:** 10.3390/ijerph19159081

**Published:** 2022-07-26

**Authors:** Kow-Tong Chen

**Affiliations:** 1 Department of Occupational Medicine, Tainan Municipal Hospital, Tainan 701, Taiwan; ktchen@mail.ncku.edu.tw; 2 Department of Public Health, College of Medicine, National Cheng Kung University, Tainan 701, Taiwan

## 1. Introduction

Emerging infectious diseases (EIDs) are defined as diseases that are newly identified, newly introduced, or newly evolved; or diseases that have recently and rapidly changed in incidence or expanded geographic, host, or vector range agents; or previous infections that acquire new virulence factors; or infections that spread to unaffected regions [1,2]. Factors associated with the emergence of these infectious agents include changes in the environment, changes in human behavior, and the emergence of antimicrobial resistance [1].

EIDs cause a huge economic crisis and public health problems in the world [3,4]. It is postulated that the origins of EIDs are significantly correlated with socio-economic, environmental, and ecological factors and provide a clue for identifying regions where new EIDs are most likely to originate [5,6]. They also present a basis for the risk of wildlife zoonotic and vector-borne EIDs, originating at lower latitudes, where reporting effort is low [6].

Previous studies have shown that the morbidity of EIDs is increasing and that most EIDs are caused by zoonoses (60.3%), the majority of which (71.8%) originate in wildlife [4,6]. Many EIDs are zoonotic; organisms grow in animals with random transmission into the human population; EID may also be foodborne, vector-borne, or air-borne, such as Spanish flu, severe respiratory syndrome coronavirus (SARS-CoV), Middle Eastern respiratory syndrome coronavirus (MERS-CoV), and severe acute respiratory syndrome coronavirus 2 (SARS-CoV-2) [7,8,9,10,11,12,13]. More than 30 human infectious pathogens have emerged in the last three decades, 75% of which spread from animals to humans through various routes [6,14]. In contrast, some pathogens that originate in humans, such as *Mycobacterium tuberculosis*, can be transmitted from humans to animals [15]. Close contact between animals and humans can increase the risk of inter-transmission. Owing to the frequent interaction between human–animal–environmental interfaces, a multi-sectoral collaboration between numerous disciplines is required to control these epidemics/pandemics [16].

The concept of one Health was initiated in multiple disciplines in 2006 [17]. One Health is a collaborative and global effort to achieve the best health for people, animals, and the environment [18]. The One Health concept aims to capture the inherent interdependence of human and non-human health and the environment and is relevant to the development of strategies to control infectious diseases [19,20]. Using a multi-sectoral and trans-disciplinary approach, EIDs threats can be better monitored and controlled. The One Health approach enhances the knowledge of zoonotic diseases and can share information about diseases between animals and humans, with the goal of achieving better health outcomes [21,22]. The relationship between animal health and human health with regard to companion animals can be described as sharing the same living environment and often treated with the same medicines if infections occur [21]. However, with the latter, the development of antibiotic resistance may play a role in the occurrence of EIDs [21].

It is suggested that the One Health approach to strengthen the surveillance of human and animal health could reduce costs by 10–30% [23]. In addition to financial resource savings, human resource investments, such as field-based healthcare workers, epidemiologists, microbiologists, infection prevention scientists, control specialists, and veterinarians, also reduce expenditure. The workforce should be well-trained, adequately equipped, and capable of rapid deployment. In addition, the timely communication of real-time epidemiological data is essential. Thus, the implementation of the One Health approach has the potential to prevent and control transboundary zoonotic diseases (such as SARS, MERS, and coronavirus infectious disease -19 (COVID-19)) efficiently and cost-effectively. The One Health approach is highly efficient in terms of resource use efficiency [24].

To prevent and control EIDs, we may need to develop more advanced systems, such as genomic precision surveillance systems, that help to detect and trace the causes of outbreaks on time [25]. Additionally, enhanced public health infrastructure and restricted international travel and trade are anticipated [12,13,14]. Environmental factors, such as patterns of land and water use, intensive livestock farming, the deterioration of wildlife habitats, the overuse of pesticides, and the international trade of wildlife, are necessary to be properly investigated and regulated in harmony with nature [26]. Intersectoral health system collaboration is required for complex monitoring and EIDs interventions. In particular, communication at regional, national, and international levels across the One Health sector limits the impact of these changes on the health of all [24].

Although there is much knowledge on the detection of pathogens and response to the occurrence of EIDs, more public health workers are needed in the field to control the spread of EIDs, care for patients with EIDs, improve effectiveness, and implement timely public health responsiveness [27]. To achieve this, the One Health approach needs to understand the biological, social, environmental, and genetic determinants of EIDs in humans and animals [28].

Recent EIDs pandemic threats have raised a public issue around resilience in health systems and their ability to react and reflect when an epidemic of EIDs occurs [29]. The contributions to this Special Issue indicate a One Health approach to the risk assessment of and response to EIDs, focusing on the interconnections between human health, animal health, and the environment. This strategy is essential for the resilience of the EIDs response system and public health systems. In addition to the public health systems for rapid detection and response, the broader One Health view urges us to rethink the efficiency of the response system. Simultaneously, we should consider the economic burden due to the occurrence of EIDs, including society, agriculture, food, international travel, and trade activities [29,30].

Although most people accept the concept of One Health, multi-sectoral cooperation in the surveillance and control of EIDs is challenging because of the significant gap between the fields of animal and human health [31]. To reduce this gap, the government needs to provide financial support to establish inter-disciplinary response structures through the One Health approach, such as establishing multi-sector systems for the interventions of EIDs arising from emerging zoonotic diseases and early warning systems of threats to humans from animals [31,32].

In addition, following the epidemic occurrence of SARS-CoV-2 on 12 December 2019 in China and its emergence as an international threat, coronavirus infectious diseases-19 (COVID-19) has rapidly spread worldwide to become a global pandemic affecting over 556.3 million confirmed cases, and over 6.3 million deaths have occurred as of 6 July 2022 [13,33]. Today, EIDs are mostly global threats to our health. They are borderless and require greater cooperation between regions and nations [32]. The COVID-19 pandemic has highlighted the urgent need for cooperation at the international level [13]. Strong international collaborations that extend beyond the borders of each country and global governance structures have been raised [32]. It is hoped that the One Health approach will gradually start at the beginning point through the education of policymakers, scientists, and citizens. To achieve this objective, as in the field of health promotion, has the responsibility to inform and alert these subjects, and participate in inducing a change in human behavior towards nature, as quickly as possible, because time is urgent [32].

In the future, there will be many challenges. The challenges of EIDs include changes in the epidemiological characteristics of EIDs infections, the transmission routes of EIDs, the density of vectors, patterns of human and non-human migration, food resources, accessibility of medical and financial sources, and other relevant factors [21,26]. However, there is an urgent need to study the epidemiological evidence on the joint effects of One Health approach strategies, the morbidity of infectious diseases among animal and human populations, preparedness and response programs for EIDs, and building an early warning system [21,22,27].

## 2. Conclusions

In summary, EIDs are a major challenge for the future. The One Health concept is a worldwide strategy that recognizes that public health concerns human health, animal health, and the environment. The One Health approach can enhance knowledge for designing effective preventive and control measures against EIDs. The development of an effective early warning system using the One Health approach will lower the economic impact of EIDs by improving the existing EIDs surveillance and preventive measures. Future research should take into account the factors among humans, animals, and the environment for a better understanding of the complex nature of transmission of EIDs, as well as for improving the prevention and control measures for EIDs. We hope this issue will bring to light the many components surrounding EIDs and promote the need for future research, collaboration, and innovative ideas to reduce the impact of contracting EIDs.
